# Inequality of Paediatric Workforce Distribution in China

**DOI:** 10.3390/ijerph13070703

**Published:** 2016-07-12

**Authors:** Peige Song, Zhenghong Ren, Xinlei Chang, Xuebei Liu, Lin An

**Affiliations:** Department of Child, Adolescent and Women’s Health, School of Public Health, Peking University, Beijing 100191, China; peigesong@hsc.pku.edu.cn (P.S.); rzhong65@126.com (Z.R.); changxl_echo@163.com (X.C.); liuxuebei87@126.com (X.L.)

**Keywords:** paediatric workforce, health human resource, inequality, China

## Abstract

Child health has been addressed as a priority at both global and national levels for many decades. In China, difficulty of accessing paediatricians has been of debate for a long time, however, there is limited evidence to assess the population- and geography-related inequality of paediatric workforce distribution. This study aimed to analyse the inequality of the distributions of the paediatric workforce (including paediatricians and paediatric nurses) in China by using Lorenz curve, Gini coefficient, and Theil L index, data were obtained from the national maternal and child health human resource sampling survey conducted in 2010. In this study, we found that the paediatric workforce was the most inequitable regarding the distribution of children <7 years, the geographic distribution of the paediatric workforce highlighted very severe inequality across the nation, except the Central region. For different professional types, we found that, except the Central region, the level of inequality of paediatric nurses was higher than that of the paediatricians regarding both the demographic and geographic distributions. The inner-regional inequalities were the main sources of the paediatric workforce distribution inequality. To conclude, this study revealed the inadequate distribution of the paediatric workforce in China for the first time, substantial inequality of paediatric workforce distribution still existed across the nation in 2010, more research is still needed to explore the in-depth sources of inequality, especially the urban-rural variance and the inner- and inter-provincial differences, and to guide national and local health policy-making and resource allocation.

## 1. Introduction

Child health has been addressed as a priority at both national and global levels for many decades [1]. Since the adoption of the Millennium Development Goals (MDGs) in September 2000, unprecedented progress has been made in improving child health and reducing child mortality all around the world [2]. The year of 2015 witnessed the end of the MDGs period and the new era of the Sustainable Development Goals (SDGs) for guiding the post-2015 development agenda. In the MDGs era, China was regarded as one of the most successful countries in reaching MDG 4, which called for a two thirds reduction of child mortality from 1990 to 2015 [2,3,4]. However, despite the national achievement, 12% of counties were still lagging behind in achieving MDG 4 by the year of 2012, the regional development across the country was heavily uneven [3].

According to the new requirements of SDG 3, continual efforts should be made to end preventable deaths of children, and to make sure that every child can have equitable access to affordable, accountable, appropriate health services of assured quality [5,6,7]. This is especially challenging for developing countries [8]. In the Chinese child development blueprint for this decade (“Outline for the Development of Chinese Children (2011–2020)”), the central government showed the determination to make health services more equitable and accessible for children by the year of 2020 [9].

Child health workforce refers to all people engaged with the primary intent of enhancing child health, including paediatricians, paediatric nurses, midwives, dentists, pharmacists, and laboratory technicians, etc. [10,11]. Worldwide, the recruitment, development, training, and retention of the health workforce is generally regarded as essential for child health improvement and has also been listed as a priority in SDG 3 for the health development in the next generation [4,12]. The efforts of the peadiatric workforce is essential for improving child health [13], however, the difficulty of accessing paediatricians has been of debate for a long time in China [14,15], although universal health insurance coverage has been achieved in 2011, and 95% of the Chinese population was insured by the end of 2011 [16], another crucial component of health system—health workforce, still remains as an obstacle in reaching universal health coverage [5,17,18,19]. However, according to the national maternal and child health human resource investigation in 2010, the overall quantity of child health human resource was sufficient to workload at both the national and regional levels [9].

This controversy may be the result of the uneven distribution of the health workforce within the country, however, there is limited evidence to support this proposition, especially for the workforce in the paediatric department where no regular national or local health workforce census is being conducted [19]. To fill this gap of knowledge, we analysed the inequality of the paediatric workforce (including paediatricians and paediatric nurses) distribution in China in this study by using the national investigation of maternal and child health human resource in 2010 [9,11].

## 2. Methods

### 2.1. Sampling Methods

**Figure 1:**
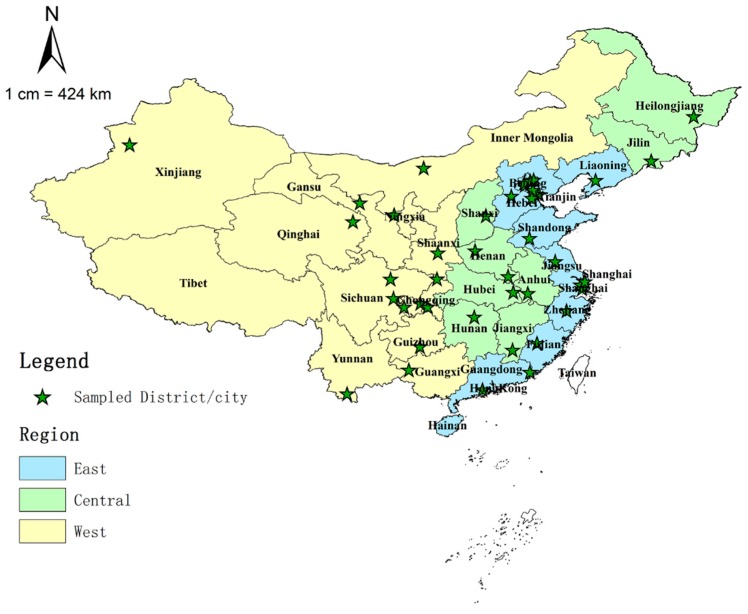
Map of China showing the East, Central, and West regions and sampled districts/cities. Notes: the three regions (East, Central, and West) were categorized according to the China Health and Family Planning Yearbook [22].

This study was a national institution-based sampling survey supported by the National Health and Family Planning Commission of the People’s Republic of China (the former Chinese Ministry of Health until 2013) [20,21]. The study design has been published and described in detail elsewhere [9,11]. Firstly, in the 22 provinces and five autonomous regions in China, 28 districts/cities were sampled from the 332 municipality prefectural districts/cities using random clustering sampling, then, in the four municipalities (Beijing, Shanghai, Tianjin, and Chongqing), two urban districts and two rural counties were drawn, respectively. Finally, there were 44 districts/cities included in this survey, all of which came from provincial-level administrative divisions in China except for Tibet Autonomous Region, Hainan province (there are only a few cities), Hong Kong Special Administrative Region, Macau Special Administrative Region, and Taiwan province, the geographic distribution of the sampled areas is shown in Figure 1.

### 2.2. Data Collection

A structured questionnaire, which was developed and improved by several rounds of expert consultation and then piloted in September 2011 before the investigation, was sent to all the medical and healthcare institutions providing maternal and child health services within the sampled districts/cities from November 2011 to February 2012. All of the data on population size, population structure, and geographic area, etc., were obtained from the local government, and the information about the number of health workers was obtained from the institutions. When counting the number of workers, according to the expert consultation, a weight of 0.5 was assigned to workers who were not working full-time to avoid overestimation of the total workforce. The total number of workers in every institution within the sampled district/city was then added together to reveal the total size of the workforce. All data was then anonymised, sent back by mail, and we were granted permission to use and analyse the data by the participating districts/cities and institutions. In this study, we only focused on the professional paediatric workforce who were serving children in the front line, namely paediatricians and paediatric nurses. All other paediatric workers providing auxiliary services (health education, training, and supervision, etc.) were not included because of their interchangeability.

### 2.3. Assessment of Inequality

Lorenz curve and Gini coefficient are commonly used to assess the inequality of the health resources distribution [23]. The Lorenz curve and Gini coefficient were first developed in the area of economics and have been widely adopted in the public health research area [19,24,25]. The x-axis of the Lorenz curve represents the cumulative share of population or geography, the y-axis shows the cumulative share of the paediatric workforce, the ideal equality distribution is shown as a diagonal line, the greater the distance from the ideal equality line, the greater the inequality [26,27]. Then the ratio of the area between the Lorenz curve and the ideal equality line was derived as the Gini coefficient. The larger the Gini coefficient, the greater the inequality. A Gini coefficient <0.2 indicates absolute equality, 0.2–0.3 relative equality, 0.3–0.4 proper inequality, 0.4–0.5 large inequality, and above 0.5 represents severe inequality [28,29]. The formula adopted in this study for calculating the Gini coefficient is:

(1) $$G\;=\;1-\overset{k-1}{\underset{i\;=\;0}{\sum }}\left(CY_{i+1}+CY_{i}\right)\left(CX_{i+1}-CX_{i}\right)$$

where G is the Gini coefficient; $CY_{i}$ is the cumulative proportion of the health workforce (paediatricians or paediatric nurses) in the ith district/city; $CX_{i}$ is the cumulative proportion of the demographic/geographic variable (number of targeted population or geographic area) in the ith district/city; and k is the total number of the districts/cities [26].

The Theil L index is a widely adopted indicator to detect the inequality and divergence with the advantage of decomposition, which can decompose the total national inequality to inner-regional difference and inter-regional difference [25,30]. The larger the Theil L index, the greater the inequality. However, the Theil L index is a relative indicator, so there is no universal assessment standard of inequality levels [30]. The formula adopted for calculating Theil L index is:

(2) L = ∑i(XiX)log⌊(XiX)/(YiY)⌋

where *L* is the Theil L index; $Y_{i}$ is the proportion of the health workforce (paediatricians or paediatric nurses) in the ith district/city; and $X_{i}$ is the proportion of the demographic/geographic variable (number of targeted population or geographic area) in the ith district/city [25].

### 2.4. Statistical Analysis

Then the numbers of paediatricians/paediatric nurses/total paediatric workforce per 10,000 population, per 10,000 children <7 years, per 10,000 children <18 years, and per 1000 live births were calculated, respectively, to reveal the demographic distribution; the numbers of paediatricians/paediatric nurses/total paediatric workforce per square kilometre were calculated, respectively, to address the geographic distribution. Then the sampled districts/cities were grouped into three regions: East, Central, and West, where the East region is the most developed area, the Central region is less developed than the East, and the West is the least developed. According to the National Health and Family Planning Commission of the People’s Republic of China, the East region includes 11 provinces: Beijing, Tianjin, Hebei, Liaoning, Shanghai, Jiangsu, Zhejiang, Fujian, Shandong, Guangdong and Hainan; the Central region includes eight provinces: Shanxi, Jilin, Heilongjiang, Anhui, Jiangxi, Henan, Hubei and Hunan; and the West region includes 12 provinces: Inner Mongolia, Guangxi, Shaanxi, Gansu, Qinghai, Ningxia, Xinjiang, Sichuan, Chongqing, Guizhou, Yunnan and Tibet (Figure 1) [22].

Finally, the corresponding Lorenz curves were drawn, the Gini coefficients and Theil L indexes were calculated for the above indicators to assess the inequality at the national and regional (East, Central, and West) levels, and the Theil L indexes were then decomposed to assess the share of inner-regional and inter-regional inequality.

The geographic distribution of the sampled districts/cities was mapped by ArcGIS 10.1 (Environmental Systems Resource Institute, Redlands, CA, USA), all analyses were conducted in SPSS 13.0 (SPSS Inc., Chicago, IL, USA), and the Lorenz curves and bar charts were drawn using Microsoft Excel 2013 (Microsoft Corporation, Redmond, WA, USA).

## 3. Results

In the 44 sampled districts/cities, there were 34,964 paediatric workers, including 16,830 (48.1%) paediatricians and 18,134 (51.9%) paediatric nurses. On average, there were 2.62 paediatric workers (including paediatricians and paediatric nurses) per 10,000 population, 34.57 per 10,000 children <7 years, 12.48 per 10,000 children <18 years, 25.27 per 1000 live births, and geographically 0.046 per square kilometre. The detailed demographic and geographic distributions of paediatric workers in the 44 sampled districts/cities are listed in Tables S1 and S2.

**Figure 2:**
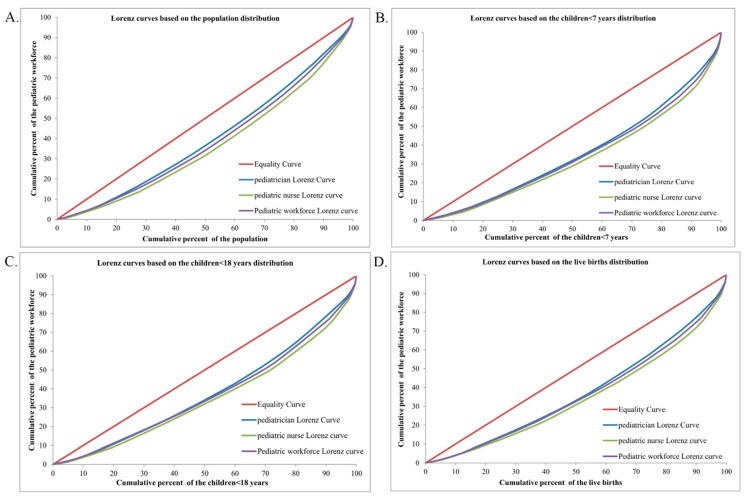
Lorenz curves for the demographic distributions of different indicators. (**A**) Based on the population distribution; (**B**) Based on the children <7 years distribution; (**C**) Based on the children <18 years distribution; (**D**) Based on the live births distribution.

**Figure 3:**
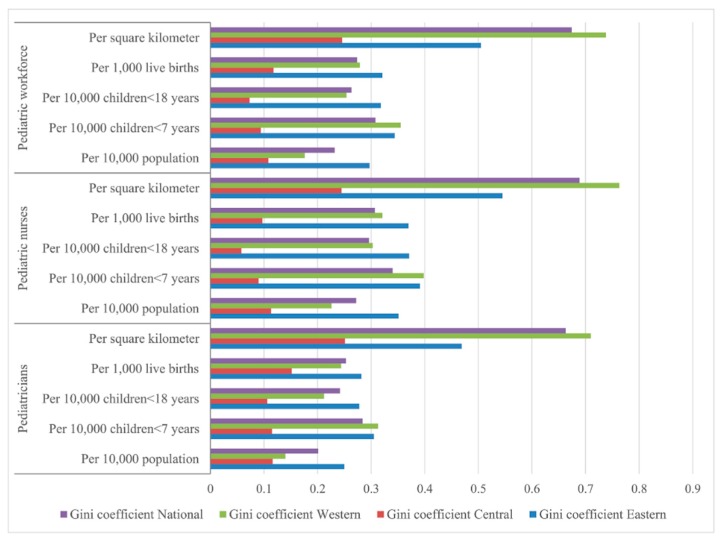
Comparison of Gini coefficients of different indicators at both national and regional levels.

**Figure 4:**
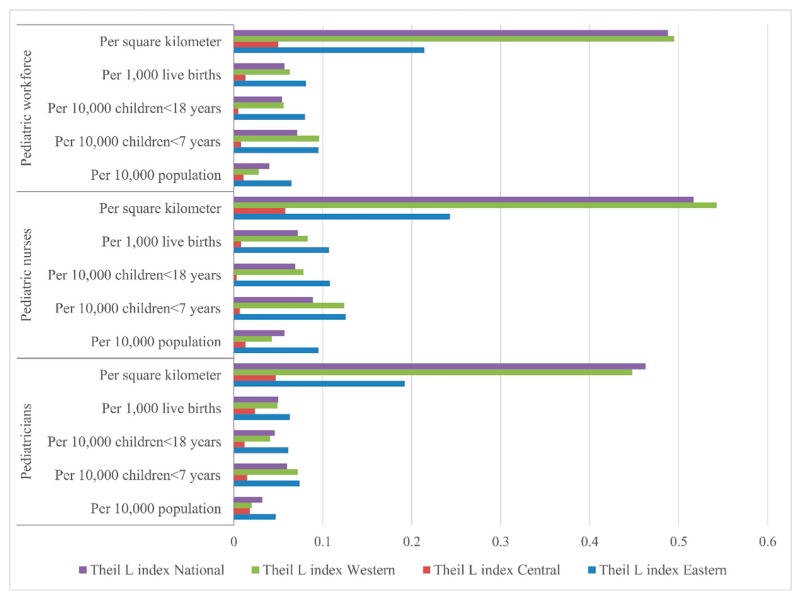
Comparison of Theil L indexes of different indicators at both national and regional levels.

**Figure 5:**
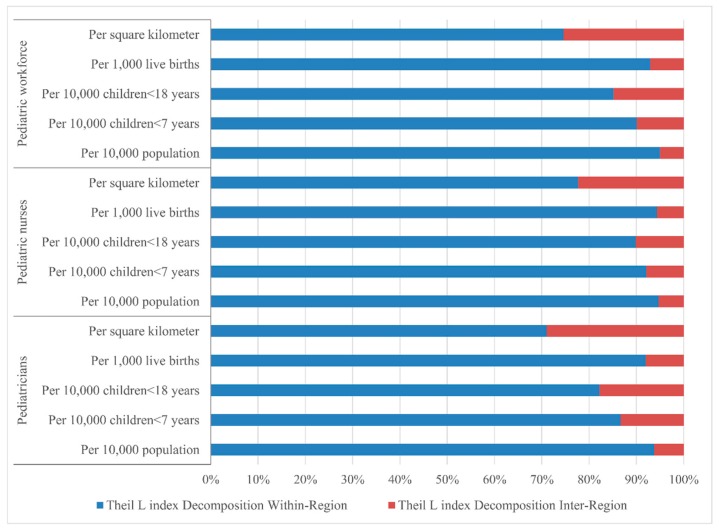
The share of inequality sources by Theil decomposition.

The Lorenz curves based on demographic distribution are presented in Figure 2, the Gini coefficients and Theil L indexes of the demographic distribution of paediatric workforce are shown in Table 1. In 2010, the distribution of the overall paediatric workforce per 10,000 population across the nation indicated a relative equality with the Gini coefficient of 0.232, among all the three regions, the most inequitable region was the East with the highest Gini coefficient (0.297) and Theil index (0.065). According to the Theil L decomposition, the majority (95.00%) of this inequality came from the inner-regional inequality, and only 5.00% was because of the inter-regional difference. The corresponding comparisons of the Gini coefficients and Theil L indexes at both national and regional levels are shown in Figure 3 and Figure 4. The distribution of paediatricians per 10,000 population was more equitable than that of paediatric nurses (Gini: 0.201 vs. 0.272, Theil L: 0.032 vs. 0.057), and both were at the level of relative equality. Similar to the distribution of the overall paediatric workforce, the East region also had the highest inequality among all three regions regarding the distributions of paediatricians and paediatric nurses, where the Gini coefficients were 0.250 and 0.351, and the Theil L coefficients were 0.047 and 0.095, respectively. The distribution of paediatric nurses in the East had reached a proper inequality level. The share of inequality sources is shown in Figure 5; 93.75% and 94.74% of the inequality of the paediatrician and paediatric nurse distributions came from inner-regional inequalities, respectively.

Concerning the serving population, the inequality of the paediatric workforce per 10,000 children (children <7 years, and children <18 years) is shown in Table 1. For younger children, the distribution of the overall paediatric workforce per 10,000 children <7 years in 2010 indicated a proper inequality (Gini = 0.308), which was lower than that in the East (Gini = 0.344) and the West (Gini = 0.355), whereas the Central region was at the level of absolute equality (Gini = 0.094). This was the same situation when being assessed by the Theil L index; the East and the West had the higher Theil L indexes of 0.095 and 0.096 respectively, and the Central had the lowest Theil L index of 0.008. According to the Theil L decomposition, the majority (90.14%) of this inequality came from the inner-regional inequality, and only 9.86% came from the inter-regional inequality. The distribution of paediatricians per 10,000 children <7 years represented a relative inequality (Gini = 0.284), but the corresponding distribution of paediatric nurses reached to the level of proper inequality with the Gini of 0.340. The East and the West showed higher inequality regarding both the distributions of paediatricians and paediatric nurses (Table 1), while the Central region indicated an absolute equality with a Gini of 0.115 and 0.090 for the paediatrician and paediatric nurse distributions, respectively. Inner-regional inequality accounted for 86.67% and 92.13% of the overall inequality of the distribution of paediatricians and paediatric nurses, respectively.

For the entire child group (children <18 years), the distribution of the overall paediatric workforce per 10,000 children <18 years in 2010 was at the level of relative equality (Gini = 0.263). As shown in Table 1 and Figure 3. Among the three regions, the East had the highest inequality (Gini = 0.318, Theil L = 0.080) and reached the level of proper inequality. The inequality of the West was lower, at the level of relative equality (Gini = 0.253), however, the Central region indicated an absolute equality with a Gini of 0.073. This was the same situation when being assessed by the Theil L index. The main source of the inequality was the inner-regional inequality, which accounted for 85.19% of the whole inequality. Nationally, the distributions of paediatricians and paediatric nurses per 10,000 children <7 years both represented a relative equality with the Gini of 0.242 and 0.296, respectively. For the distribution of paediatricians per 10,000 children <18 years, the East and the West were at the level of relative equality, but the East was more inequitable than the West (Gini: 0.278 vs. 0.212, Theil L: 0.061 vs. 0.041), the Central region indicated an absolute equality with a Gini of 0.106. The main inequality (80.43%) came from inner-regional inequality. For the distribution of paediatric nurses per 10,000 children <18 years, the East and the West reached the level of proper inequality with a Gini of 0.371 and 0.303 respectively, but the Central region showed an absolute equality. Inner-regional inequality accounted 89.86% of this inequality.

When concerning about the future serving population (live births), the distribution of the overall paediatric workforce per 1000 live births in 2010 indicated a relative equality (Gini = 0.274), the East reached to the level of proper inequality (Gini = 0.321), and the West indicated a relative equality (Gini = 0.279), the Central region was at the lowest level of inequality with a Gini of 0.118. According to the Theil L decomposition, the majority (92.98%) of this inequality came from the inner-regional inequality, and only 7.02% came from the inter-regional inequality. The distribution of paediatricians per 1000 live births represented a relative equality (Gini = 0.253), but the corresponding distribution of paediatric nurses reached to the level of proper inequality with a Gini of 0.307. The East and the West both showed higher inequality regarding the distributions of paediatricians and paediatric nurses, while the Central region indicated an absolute equality with a Gini of 0.152 and 0.097 for the paediatrician and paediatric nurse distributions, respectively. Inner-regional inequality accounted for 92.00% and 93.06% of the overall inequality of the distribution of paediatricians and paediatric nurses respectively.

**Figure 6:**
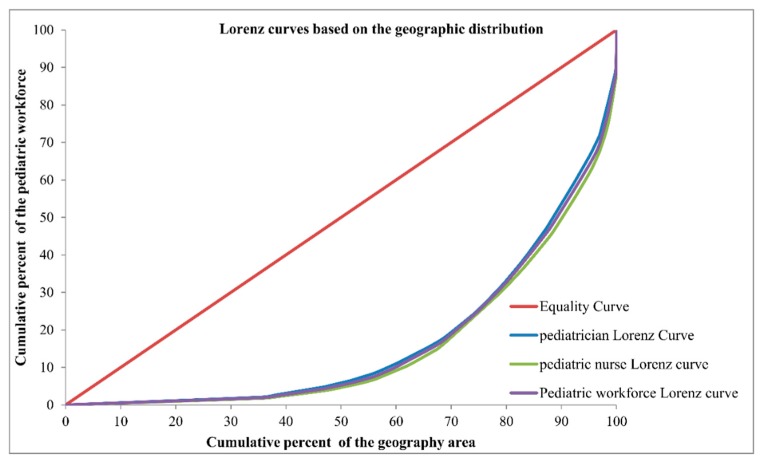
Lorenz curves for the geographic distributions of different indicators.

The Lorenz curve based on geographic distribution of the paediatric workforce is presented in Figure 6. The Gini coefficients and Theil L indexes of the geographic distribution of the paediatric workforce are shown in Table 1. The national distribution of the paediatric workforce per square kilometre indicated a severe inequality (Gini = 0.674), the most inequitable was the West region with a very high Gini of 0.738; the East was also in the level of severe inequality, but with a lower Gini of 0.505, whereas the Central region represented a relative equality (Gini = 0.246). According to the Theil L decomposition, this inequality mainly came from the inner-regional inequality (94.59%). The distribution of paediatricians per square kilometre was less inequitable than the distribution of paediatric nurses (Gini: 0.663 vs. 0.689), and both were at the level of severe inequality. The West was the most severe inequitable region, regarding both the distributions of paediatricians and paediatric nurses among all three regions. The distribution of paediatricians in the East was at the level of large inequality (Gini = 0.469), whereas the distribution of paediatric nurses indicated a severe inequality (Gini = 0.545). The Central region was the most equitable region, regarding both the distributions of paediatricians and paediatric nurses with lower Ginis of 0.251 and 0.245, respectively. Inner-region inequality accounted for 71.06% and 77.56% of the inequality of the paediatrician and paediatric nurse distributions , respectively.

## 4. Discussion

This study is the first study with the attempt to assess the inequality of paediatric workforce distribution in China. In this study, the demographic- and geographic-related inequality of paediatric workforce distribution was examined and assessed by adopting the data of the paediatric workforce from the national representative maternal and child health human resource investigation.

Health services should be provided to the entire society [31]; this is also true for another core component of health system—the health workforce [32]. In this study, the demographic distribution of the paediatric workforce was found to be the most inequitable regarding the distribution of children <7 years; the real situation may be worse because younger children are more vulnerable to diseases, especially to communicable diseases and, thus, require more intensive health care. The indicator of live births was adopted as an approximate proxy of future children, the level of inequality of the paediatric workforce distribution to future children was higher than to the serving children in 2010, and this indicates that the inequitable distribution of the paediatric workface may be worse in the years following 2010, especially for the most developed East region. The geographic distribution of health resources is also considered as an important health policy issue in many countries [33]. In this study, the geographic distribution of the paediatric workforce highlighted very severe inequality, except in the Central region, although the population does not equally distribute according to the geographic area in the real world, especially for a vast country like China. However, the significant geographic inequality still suggested that some children would have to travel longer distance to receive paediatric services, and more measures should be taken to attract paediatric workforce in less densely populated areas [34].

For different professional types, our study indicated that, except for the Central region, the level of inequality of paediatric nurses was higher than that of paediatricians regarding both the demographic and geographic distributions. This finding is partly in line with another study exploring the workforce inequality of the Urban Community Health Service in China, where the inequality measured by nurses per 10,000 population ratio was greater than that measured by doctors per 10,000 population ratio [34]. The possible reasons might be the shortage of nurses. In addition, nurses generally have more flexible career options to stay in high-paid cities or urban areas than doctors in China, and as this may also aggravate this inequality situation [35], more priority should be given to the reasonable allocation of paediatric nurses in future health plan.

As is well known, overall inequality can be divided into the inner-group and inter-group inequality, the decomposition of inequality can be used as the first step to identify the proximate sources of inequality [25,36]. In this study, the moderately-developing Central region was found to be more equitable than the West and the East regions regarding both the demographic and geographic distributions of the paediatric workforce. This may indicate the severe inner-regional unreasonable allocation of paediatric resources within the regions [37]. In the West region, the paediatric workforce showed the highest inequality regarding the distribution of children <7 years and geography. This might be associated with the low economic development in these provinces [37]. By Theil L decomposition, this study shows that the inner-regional inequality was the main source of the paediatric workforce distribution inequality. This disparity may stem from the development inequality and related health policy within each region, and more policy attention should be paid to the inequality locally. The proportion of inter-regional inequality of paediatric nurse distribution was larger than that of paediatrician distribution, both demographically and geographically; equalization of paediatric nurses should receive greater priority across the three regions.

It is worth pointing out that both the demographic and geographic approaches are useful for providing a glance of the inequality of paediatric workforce allocation. Although the geographic distribution of the paediatric workforce reached severe inequality in China in 2010, especially in the West region, we cannot arbitrarily conclude that the paediatric workforce distribution in China was in a very urgent severe situation. For policy-makers, it is especially imperative to consider both the demographic and geographic distributions and all influencing factors when making the health allocation plans.

There are also some limitations in this study. Due to the survey design, we can only assess the inequality at the national and regional level, but not at the provincial level and, subsequently, the inequality source at the provincial level cannot be captured, either to the rural and urban difference which might be another main source of inequality, in addition, the lack of detailed basic epidemiological indices might also limit the exploration of potential sources of inequality. In the investigation, the classification of peadiatricians and peadiatric nurses were according to the professionals’ certificate types, which may not be consistent with their real work contents and, thus, limits the comparison with other similar studies from other countries. In this study, although we proposed different alternative indicators of general and child population as the surrogates of the total number of population and presented the demographic distribution of paediatric workforce from different aspects, however, the use of number of live births as the proxy of future serving population may still be imperfect, only the number of live births in one year of 2010 cannot fully represent the capacity of future children. When assessing the inequality regarding the demographic distribution, only the resident population was considered as the targeted population in this study, this assessment of inequality may influence the reflection of the real situation because of the movement of migrant workers into richer developed areas [38].

## 5. Conclusions

This study revealed the inadequate distribution of the paediatric workforce issue in China for the first time, showing that substantial inequality of paediatric workforce distribution still existed in 2010. It is suggested that the findings in this study merit making future regional health policies at the national level. However, more research is still needed to explore the in-depth sources of inequality, especially the urban-rural variance and the inner- and inter-provincial differences, and to guide national and local health policy-making and resource allocation.

## Figures and Tables

**Table 1:** Gini coefficients and Theil L index of different indicators in the three regions.

Workforce	Variable	Gini Coefficient				Theil L Index				Decomposition	
		East	Central	West	National	East	Central	West	National	Inner-Region (%)	Inter-Region (%)
Paediatricians	Per 10,000 population	0.250	0.116	0.140	0.201	0.047	0.018	0.020	0.032	0.030 (93.75)	0.002 (6.25)
	Per 10,000 children <7 years	0.305	0.115	0.313	0.284	0.074	0.015	0.072	0.060	0.052 (86.67)	0.008 (13.33)
	Per 10,000 children <18 years	0.278	0.106	0.212	0.242	0.061	0.012	0.041	0.046	0.037 (80.43)	0.008 (17.39)
	Per 1000 live births	0.282	0.152	0.244	0.253	0.063	0.024	0.049	0.050	0.046 (92.00)	0.004 (8.00)
	Per square kilometre	0.469	0.251	0.710	0.663	0.192	0.047	0.448	0.463	0.329 (71.06)	0.134 (28.94)
Paediatric nurses	Per 10,000 population	0.351	0.113	0.226	0.272	0.095	0.013	0.043	0.057	0.054 (94.74)	0.003 (5.26)
	Per 10,000 children <7 years	0.391	0.090	0.398	0.340	0.126	0.007	0.124	0.089	0.082 (92.13)	0.007 (7.87)
	Per 10,000 children <18 years	0.371	0.058	0.303	0.296	0.108	0.003	0.078	0.069	0.062 (89.86)	0.007 (10.14)
	Per 1000 live births	0.370	0.097	0.321	0.307	0.107	0.008	0.083	0.072	0.067 (93.06)	0.004 (5.56)
	Per square kilometre	0.545	0.245	0.763	0.689	0.243	0.058	0.543	0.517	0.401 (77.56)	0.115 (22.24)
Paediatric workforce	Per 10,000 population	0.297	0.108	0.176	0.232	0.065	0.011	0.028	0.040	0.038 (95.00)	0.002 (5.00)
	Per 10,000 children <7 years	0.344	0.094	0.355	0.308	0.095	0.008	0.096	0.071	0.064 (90.14)	0.007 (9.86)
	Per 10,000 children <18 years	0.318	0.073	0.254	0.263	0.080	0.005	0.056	0.054	0.046 (85.19)	0.008 (14.81)
	Per 1000 live births	0.321	0.118	0.279	0.274	0.081	0.013	0.063	0.057	0.053 (92.98)	0.004 (7.02)
	Per square kilometre	0.505	0.246	0.738	0.674	0.214	0.050	0.495	0.488	0.364 (74.59)	0.124 (25.41)

Notes: the three regions (East, Central, and West) were categorized according to the China Health and Family Planning Yearbook [22].

## Supplementary Materials

The following are available online at www.mdpi.com/1660-4601/13/7/703/s1, Table S1: The demographic distribution of paediatric workforce in the sampled districts/cities; Table S2: The geographic distribution of paediatric workforce in the sampled districts/cities.

## Abbreviations

The following abbreviations are used in this manuscript:

MDGs	Millennium Development Goals
SDGs	Sustainable Development Goals
