# Supplementary material for: Inequality of Paediatric Workforce Distribution in China
Source: Int J Environ Res Public Health. 2016 Jul 12;13(7):703. doi: 10.3390/ijerph13070703 (PMC4962244; doi:10.3390/ijerph13070703)
Supplement: Supplementary file 1 [file ijerph-13-00703-s001.pdf]

Supplementary Materials: Inequality of Paediatric Workforce Distribution in China

Peige Song, Zhenghong Ren, Xinlei Chang, Xuebei Liu and Lin An

Table S1. The demographic distribution of the paediatric workforce in the sampled districts/cities.

Region	Province	Sampled District/City	Number of Paediatricians				Number of Paediatric Nurses				Number of Paediatric Workforce			
			/10,000 Population	/10,000 Children <7 years	/10,000 Children <18 years	/1000 Live Births	/10,000 Population	/10,000 Children <7 years	/10,000 Children <18 years	/1000 Live Births	/10,000 Population	/10,000 children <7 years	/10,000 Children <18 years	/1000 Live Births
East	Beijing	Xicheng	4.34	78.18	38.31	67.50	4.70	84.59	41.45	73.02	9.04	162.77	79.77	140.52
	Beijing	Haidian	0.61	16.33	5.17	11.88	0.66	17.46	5.53	12.71	1.27	33.79	10.70	24.60
	Beijing	Shunyi	0.83	21.94	6.61	12.99	0.44	11.72	3.53	6.94	1.28	33.65	10.14	19.93
	Beijing	Pinggu	0.87	22.38	6.11	12.37	1.03	26.73	7.30	14.78	1.90	49.11	13.42	27.15
	Tianjin	Hexi	5.39	173.42	52.10	108.14	7.42	238.87	71.77	148.95	12.81	412.29	123.87	257.09
	Tianjin	Dongli	0.37	9.04	2.51	5.50	0.12	2.88	0.80	1.75	0.48	11.92	3.31	7.25
	Tianjin	Wuqing	0.65	10.50	3.77	9.08	0.22	3.56	1.28	3.07	0.87	14.06	5.04	12.15
	Tianjin	Jinghai	0.70	6.65	3.09	6.34	0.48	4.58	2.13	4.36	1.17	11.24	5.23	10.70
	Hebei	Baoding	1.06	12.32	4.95	8.23	1.10	12.76	5.13	8.53	2.17	25.08	10.08	16.77
	Liaoning	Yingkou	0.95	20.02	6.42	15.20	0.82	17.32	5.56	13.16	1.77	37.34	11.98	28.36
	Shanghai	Yangpu	1.84	47.93	14.15	40.19	2.91	75.97	22.43	63.71	4.74	123.90	36.58	103.90
	Shanghai	Minhang	2.29	89.95	18.67	35.45	3.26	127.90	26.55	50.41	5.55	217.86	45.23	85.86
	Shanghai	Jiading	0.68	7.27	8.28	13.93	0.35	3.71	4.22	7.11	1.03	10.98	12.50	21.04
	Shanghai	Chongming	0.85	20.52	7.92	17.96	0.85	20.52	7.92	17.96	1.71	41.05	15.85	35.92
	Jiangsu	Huaian	0.96	15.46	4.37	8.70	0.73	11.73	3.32	6.60	1.68	27.18	7.69	15.30
	Zhejiang	Jinhua	1.11	19.59	6.00	12.80	0.65	11.56	3.54	7.55	1.76	31.15	9.54	20.35
	Fujian	Sanming	1.31	17.11	6.49	10.70	1.35	17.63	6.68	11.02	2.66	34.75	13.17	21.72
	Shandong	Jining	1.77	22.16	8.64	14.69	1.61	20.25	7.89	13.43	3.38	42.42	16.53	28.12
	Guangdong	Jiangmen	1.46	24.20	7.50	16.18	1.67	27.70	8.59	18.52	3.13	51.90	16.09	34.70
	Guangdong	Chaozhou	0.75	9.04	3.09	6.64	0.79	9.49	3.25	6.98	1.54	18.53	6.34	13.62
Central	Shanxi	Jinzhong	1.05	14.22	4.78	10.46	0.78	10.63	3.58	7.82	1.83	24.85	8.36	18.29
	Jilin	Baishan	1.42	37.82	9.70	26.08	1.10	29.39	7.54	20.27	2.52	67.21	17.25	46.34
	Heilongjiang	Qitaihe	1.20	25.01	7.30	16.91	1.02	21.37	6.24	14.45	2.22	46.38	13.54	31.36
	Anhui	Anqing	1.16	12.75	5.31	11.95	1.08	11.90	4.96	11.15	2.23	24.65	10.26	23.10
	Jiangxi	Ganzhou	1.24	13.10	4.24	9.37	1.50	15.86	5.13	11.35	2.75	28.97	9.37	20.72
	Henan	Louyang	1.33	14.27	5.26	12.37	1.38	14.88	5.48	12.89	2.71	29.15	10.74	25.26
	Henan	Xinyang	1.22	10.72	4.28	10.02	1.35	11.86	4.73	11.09	2.57	22.58	9.01	21.11
	Hubei	Huanggang	0.92	12.48	4.17	8.34	1.07	14.63	4.89	9.77	1.99	27.11	9.06	18.11
	Hunan	Yiyang	0.44	6.45	2.39	3.83	0.83	12.16	4.50	7.22	1.27	18.61	6.88	11.05

Table S1. *Cont.*

Region	Province	Sampled District/City	Number of Paediatricians				Number of Paediatric Nurses				Number of Paediatric Workforce			
			/10,000 Population	/10,000 Children <7 years	/10,000 Children <18 years	/1000 Live Births	/10,000 Population	/10,000 Children <7 years	/10,000 Children <18 years	/1000 Live Births	/10,000 Population	/10,000 children <7 years	/10,000 Children <18 years	/1000 Live Births
West	Inner Mongolia	Bayannaoer	2.08	47.22	12.51	28.43	1.75	39.75	10.53	23.94	3.84	86.97	23.04	52.37
	Guangxi	Baise	1.76	17.25	6.94	12.66	2.07	20.34	8.18	14.93	3.83	37.60	15.12	27.59
	Chongqing	Nanan	1.03	14.31	4.58	14.47	1.17	16.32	5.22	16.51	2.20	30.63	9.80	30.98
	Chongqing	Yongchuan	0.79	11.85	3.55	7.36	0.65	9.80	2.94	6.09	1.44	21.64	6.48	13.45
	Chongqing	Qijiang	0.66	22.05	9.95	7.01	1.41	47.02	21.22	14.94	2.07	69.08	31.17	21.95
	Chongqing	Chengkou	1.55	10.64	2.76	10.97	0.78	5.32	1.38	5.48	2.33	15.96	4.14	16.45
	Sichuan	Mianyang	1.53	27.37	8.23	17.67	1.42	25.51	7.66	16.46	2.95	52.88	15.89	34.13
	Sichuan	Ziyang	1.29	8.13	5.96	13.16	1.13	7.12	5.22	11.52	2.43	15.25	11.17	24.68
	Guizhou	Qiannan	0.63	6.36	2.22	4.38	0.57	5.76	2.01	3.97	1.20	12.12	4.22	8.35
	Yunnan	Xishuangbanna	0.90	10.12	3.99	7.76	0.60	6.75	2.66	5.17	1.50	16.87	6.65	12.93
	Shaanxi	Xi'an	1.61	31.60	8.98	19.76	2.45	48.25	13.72	30.17	4.06	79.85	22.70	49.92
	Gansu	Jinchang	1.55	23.22	8.09	18.61	1.47	21.93	7.64	17.58	3.02	45.15	15.73	36.19
	Qinghai	Xining	1.45	24.46	6.63	14.42	2.25	37.92	10.27	22.35	3.69	62.38	16.90	36.77
	Ningxia	Zhongwei	1.39	15.83	4.62	10.98	1.37	15.62	4.56	10.83	2.76	31.45	9.19	21.81
	Xinjiang	Yili	1.41	15.04	5.99	8.51	1.30	13.79	5.49	7.80	2.71	28.83	11.48	16.31

Table S2. The geographic distribution of the paediatric workforce in the sampled districts/cities.

Region	Province	Sampled District /City	Number of Paediatricians /Square Kilometre	Number of Paediatric Nurses /Square Kilometre	Number of Paediatric Workforce /Square Kilometre
East	Beijing	Xicheng	5.779	6.252	12.032
	Beijing	Haidian	0.467	0.499	0.966
	Beijing	Shunyi	0.072	0.038	0.110
	Beijing	Pinggu	0.033	0.040	0.073
	Tianjin	Hexi	12.676	17.459	30.135
	Tianjin	Dongli	0.048	0.015	0.063
	Tianjin	Wuqing	0.039	0.013	0.053
	Tianjin	Jinghai	0.030	0.021	0.051
	Hebei	Baoding	0.054	0.056	0.109
	Liaoning	Yingkou	0.042	0.037	0.079
	Shanghai	Yangpu	3.976	6.303	10.279
	Shanghai	Minhang	1.499	2.131	3.629
	Shanghai	Jiading	0.215	0.110	0.325
	Shanghai	Chongming	0.053	0.053	0.105
	Jiangsu	Huaian	0.046	0.035	0.080
	Zhejiang	Jinhua	0.054	0.032	0.086
	Fujian	Sanming	0.014	0.015	0.029
	Shandong	Jining	0.128	0.117	0.244
	Guangdong	Jiangmen	0.068	0.078	0.146
	Guangdong	Chaozhou	0.054	0.057	0.111
Central	Shanxi	Jinzhong	0.021	0.016	0.036
	Jilin	Baishan	0.011	0.008	0.019
	Heilongjiang	Qitaihe	0.018	0.015	0.033
	Anhui	Anqing	0.045	0.042	0.087
	Jiangxi	Ganzhou	0.026	0.032	0.058
	Henan	Louyang	0.057	0.059	0.116
	Henan	Xinyang	0.039	0.044	0.083
	Hubei	Huanggang	0.032	0.038	0.070
	Hunan	Yiyang	0.016	0.029	0.045

Table S2. *Cont.*

Region	Province	Sampled District /City	Number of Paediatricians /Square Kilometre	Number of Paediatric Nurses /Square Kilometre	Number of Paediatric Workforce /Square Kilometre
West	Inner Mongolia	Bayannaoer	0.005	0.005	0.010
	Guangxi	Baise	0.017	0.020	0.037
	Chongqing	Nanan	0.296	0.338	0.635
	Chongqing	Yongchuan	0.051	0.043	0.094
	Chongqing	Qijiang	0.019	0.041	0.060
	Chongqing	Chengkou	0.009	0.005	0.014
	Sichuan	Mianyang	0.035	0.032	0.067
	Sichuan	Ziyang	0.060	0.052	0.112
	Guizhou	Qiannan	0.008	0.007	0.015
	Yunnan	Xishuangbanna	0.005	0.003	0.009
	Shaanxi	Xi'an	0.135	0.206	0.340
	Gansu	Jinchang	0.008	0.007	0.015
	Qinghai	Xining	0.042	0.065	0.107
	Ningxia	Zhongwei	0.010	0.010	0.020
	Xinjiang	Yili	0.001	0.001	0.003

© 2016 by the authors; licensee MDPI, Basel, Switzerland. This article is an open access article distributed under the terms and conditions of the Creative Commons by Attribution (CC-BY) license (http://creativecommons.org/licenses/by/4.0/).
